# A Natural Compound Mixture Containing Arctigenin, Hederagenin, and Baicalein Alleviates Atopic Dermatitis in Mice by Regulating HPA Axis and Immune Activity

**DOI:** 10.1155/2020/1970349

**Published:** 2020-06-29

**Authors:** Ly Thi Huong Nguyen, Tae-Woo Oh, Uy Thai Nguyen, Min-Jin Choi, In-Jun Yang, Heung-Mook Shin

**Affiliations:** ^1^Department of Physiology, College of Korean Medicine, Dongguk University, Gyeongju 38066, Republic of Korea; ^2^Korean Medicine (KM)-Application Centre, Korea Institute of Oriental Medicine (KIOM), Daegu 41062, Republic of Korea

## Abstract

Forsythiae Fructus, Lonicerae Flos, and Scutellariae Radix are medicinal herbs that possess anti-inflammatory and anti-atopic effects. Hence, we investigated the effects of a mixture (ADM), containing arctigenin, hederagenin, and baicalein, which are the main compound from these herbs on atopic dermatitis (AD) skin lesions and the underlying molecular mechanisms. ADM was topically applied to dorsal skin lesions of 2,4-dinitrochlorobenzene- (DNCB-) induced ICR mice, and the expressions of proinflammatory mediators and HPA axis hormones were investigated. The topical application of 0.5% ADM significantly reduced the DNCB-induced symptoms of AD in ICR mice. Histological analysis showed that ADM exerted antiatopic effects by reducing the epidermal thickness and mast cell infiltration into skin lesions. 0.5% ADM attenuated the levels of TNF-*α*, IFN-*γ*, IL-4, and VEGF in skin lesions and serum IgE. The production of Th1-/Th2-related cytokines in splenic tissues, including TNF-*α*, IFN-*γ*, IL-12, and IL-4, were also decreased by ADM treatment. ADM diminished corticotropin-releasing hormone (CRH), adrenocorticotropic hormone (ACTH), and corticosteroid (CORT) production in DNCB-induced mice. In vitro, ADM reduced the productions of TARC/CCL17, MDC/CCL22, IL-6, and ICAM-1 in TNF-*α*/IFN-*γ*- (TI-) stimulated HaCaT cells by suppressing the ERK and JNK signaling pathways. In addition, ADM inhibited corticotropin-releasing hormone/substance P- (CRH/SP-) induced VEGF production in HMC-1 cells. These results suggest that ADM may have therapeutic potential in AD by reducing inflammation and attenuating HPA axis activation.

## 1. Introduction

Atopic dermatitis (AD) is the most common chronic relapsing inflammatory skin disorder and is characterized by severe pruritus and eczematous and lichenified skin lesions. AD affects 15–20% of the world's population, and its prevalence continues to increase, especially in industrialized countries [1]. Keratinocytes are the main cell type in skin epidermis and participate in the development of AD. Under normal conditions, keratinocytes form a mechanical defense against the external environment and prevent allergic sensitization to antigens. Keratinocytes maintain their barrier function by modulating proliferation/differentiation processes and by producing cytokines and chemokines [2]. However, in cases of AD, epidermal barrier impairment increases the absorptions of external toxins and promotes infection, which leads to immune abnormalities [3]. Keratinocytes in skin lesions from AD patients can produce thymus and activation-regulated chemokine (TARC)/CCL17 and macrophage-derived chemokine (MDC)/CCL22, which are chemoattractive for T-helper (Th) 2 cells [4], and when these cells infiltrate skin lesions, they release various inflammatory cytokines, such as interleukin (IL)-4, IL-5, IL-10, and IL-13. These cytokines may then activate the secretions of immunoglobulin E (IgE) and histamine by immune cells, which are important in the progress of allergic diseases, including AD [5]. Furthermore, during the pathogenesis of AD, immune cell infiltration leads to skin barrier dysfunction, disrupts skin immune response, exacerbates inflammation, and thus establishes a vicious cycle.

Accumulating evidence indicates that AD patients exhibit elevated levels of stress and anxiety [6, 7]. Interestingly, AD patients also exhibit impaired hypothalamic-pituitary-adrenal (HPA) axis responses and excessive sympathetic-adrenal medullary responses to psychological stress [8]. The HPA axis is a crucial neuroendocrine system that is responsible for communication between the brain and skin. Corticotropin-releasing hormone (CRH) is released from the hypothalamus and stimulates the pituitary to secrete adrenocorticotropic hormone (ACTH) release to the blood, which then triggers glucocorticoid (GC) (cortisol in humans and corticosterone in rodents) release from the adrenal cortex. High concentrations of GC can cause neuronal damage, decreased neurogenesis, and apoptosis in the hippocampus, which is involved in stress and anxiety [9]. In the skin, mast cells are important players in the pathogenesis of AD and can be activated by stress hormones like CRH. Previous studies have suggested CRH might activate mast cells to release pro-inflammatory mediators, such as IL-8, tumor necrosis factor (TNF), and vascular endothelial growth factor (VEGF), which are known to contribute to skin inflammation in AD [10]. VEGF stimulates angiogenesis, which is the hallmark feature of atopic dermatitis that is linked to the inflammatory process. A clinical study correlated the VEGF serum levels with the severity of AD [11]. Therefore, VEGF is an important mediator in the pathogenesis of AD.

**Figure 1:**
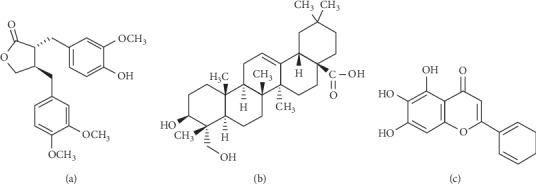
Chemical structures of (a) arctigenin, (b) hederagenin, and (c) baicalein.

The most important therapy for AD is a topical corticosteroid application, but the long-term use of corticosteroids has a variety of adverse side effects, which include epidermal barrier dysfunction and immunosuppression [12]. Thus, new therapeutic agents with few side effects are required for the treatment of AD. Forsythiae Fructus, the unripe fruit of *Forsythia viridissima* Lindl., is used to treat erysipelas, skin rash, and acute and chronic inflammatory disorders. The efficacy of Forsythiae Fructus has been suggested to be due to the inhibition of mast-cell-mediated allergic inflammatory reactions [13]. Lonicerae Flos, the dried buds of *Lonicera japonica* Thunb, is indicated for the treatment of carbuncles disease, pharyngitis, and erysipelas and has been demonstrated to exhibit antiatopic dermatitis effects in an NC/Nga mouse model of AD [14]. Scutellariae Radix, the dried root of the medicinal plant *Scutellariae baicalensis* Georgi., has been shown to modulate the immune response in AD by suppressing the productions of IL-5 and IL-10 [15]. Since these herbs possess therapeutic anti-inflammatory effects, we hypothesized that a combination of compounds derived from these herbs might be effective in AD. In the present study, we investigated the effects of a mixture of three natural compounds (ADM), including arctigenin (from Forsythiae Fructus), hederagenin (from Lonicerae Flos), and baicalein (from Scutellariae Radix) (Figure 1), in vitro and in vivo on AD-like factors and AD-induced stress-related hormones.

## 2. Materials and Methods

### 2.1. Chemicals and Reagents

Arctigenin, hederagenin, and baicalein were purchased from Chemfaces (Hubei, China). 2,4-Dinitrochlorobenzene (DNCB), corticotropin-releasing hormone CRH, substance P (SP), 1-thioglycerol, U0126, SB202190, and SP600125 were obtained from Sigma-Aldrich (St. Louis, MO, USA). High-glucose Dulbecco's modified Eagle's medium (DMEM) was obtained from Welgene Inc. (Gyeongsangbuk, Korea). Iscove's modified Dulbecco's medium (IMDM) was purchased from Merck Millipore (Darmstadt, Germany). Fetal bovine serum (FBS) and antibiotics were obtained from Invitrogen Inc. (Carlsbad, CA, USA). Human IL-6, ICAM-1, VEGF, and mouse IL-13, TNF-*α*, IFN-*γ*, IL-12, VEGF, and IgE ELISA kits were purchased from Koma Biotech Inc. (Seoul). Human TARC/CCL17, MDC/CCL22, and mouse IL-4 ELISA kits were obtained from R&D Systems (Minneapolis, MN, USA). Antibodies for phosphorylated extracellular signal-regulated kinase (p-ERK), phosphorylated c-Jun N-terminal kinase (p-JNK), and phosphorylated p38 (p-p38) were purchased from Cell Signaling Technology (Danvers, MA, USA). HRP-conjugated anti-*β*-actin was obtained from Sigma-Aldrich (St. Louis, MO, USA).

### 2.2. Animals and Treatment

ICR (5-week-old male) mice were purchased from Koatech (Gyeonggi, Korea). All animal experimental procedures were conducted in accordance with the protocols approved by the Institutional Animal Care and Use Committee of Dongguk University (Approval No. IACUC-2018-011). Mice were randomly divided into five groups (*n* = 5): a normal control group (NC), DNCB only group (DNCB), DNCB plus treatment with a low dose of ADM (ADM 0.05%), DNCB plus treatment with a high dose of ADM (ADM 0.5%), and DNCB plus treatment with 0.1% dexamethasone group (DEX). Animals were acclimated for one week before experiments. Briefly, backs of mice were shaved, and 200 *μ*l of 1% DNCB (dissolved in a 3 : 1 mixture of acetone and olive oil) was applied three times during the first week (the sensitization period). From the second week, 200 *μ*l of 0.2% DNCB was applied to dorsal skin three times per week for six weeks. After the induction of AD-like skin lesions, dorsal skins of appropriate animals were treated with 200 *μ*l ADM (0.05% or 0.5%) or DEX (0.1%) daily for a week. ADM was prepared by dissolved equal amounts of each compound in a 3 : 1 mixture of acetone and olive oil to concentrations of 0.05% and 0.5%. For example, “ADM 0.5%” means a composition containing ≈ 1.67 mg/ml of arctigenin, hederagenin, and baicalein, respectively. The day after the final treatment, mice were sacrificed, whole blood was collected, and serum was obtained by centrifugation (3,000 ×g, 15 min, 4°C). Body and spleen weights were recorded. Dorsal skin lesions and brain tissues were excised and kept in 4% paraformaldehyde for histological analysis or homogenized using tissue extraction reagent for protein expression analysis.

### 2.3. Histological Analysis

Dorsal skins were fixed in 4% paraformaldehyde and embedded in paraffin. Sections were cut and stained with hematoxylin and eosin (H&E) or toluidine blue (TB) to detect mast cell infiltration. Histological analysis was performed using an optical microscope (Olympus CK40–32PH, Japan) and DIXI image solution 2.89 software (DIXI Optics, Daejeon, South Korea).

### 2.4. Histopathological Assessment

Brain sections were deparaffinized in xylene, sequentially rehydrated in graded ethanol, immersed in 0.01 M PBS (pH 7.4), microwaved for 5 min in 0.01 M sodium citrate buffer (pH 6.0), cooled to room temperature, and washed three times for 3 min in PBS. They were then incubated in 3% hydrogen peroxide for 20 min to eliminate endogenous peroxidase activity, washed in PBS, stained with H&E, or 0.1% cresyl violet (for Nissl staining). Images were captured using a Nikon fluorescence microscope equipped with NIS-Elements BR 4.50 software (Nikon, Tokyo).

### 2.5. Cell Culture and Treatments

HaCaT cells (a human keratinocyte cell line) were cultured in DMEM supplemented with 10% FBS and 1% penicillin-streptomycin at 37°C in a 5% CO_2_ humidified atmosphere. The medium was changed every two days during incubation. Cells were made quiescent by starvation in serum-free medium for 24 h, pretreated with ADM (10, 30 *μ*M) or DEX (10 *μ*M) for 1 h, and then stimulated with TI (TNF-*α*/IFN-*γ*, 10 ng/ml of each) for the indicated times. ADM was prepared by mixing the three compounds at a concentration of 10 *μ*M each in DMSO. For example, “ADM 30 *μ*M” means a composition containing 10 *μ*M of arctigenin, hederagenin, and baicalein, respectively.

HMC-1 cells (a human mast cell-line) were obtained from Merck Millipore (Darmstadt, Germany) and cultured in IMDM supplemented with 10% FBS, 1% penicillin-streptomycin, 1.2 mM 1-thioglycerol, at 37°C in 5% CO_2_ humidified atmosphere. HMC-1 cells were incubated with 10 *μ*M SP for 48 h. Then, ADM (10 or 30 *μ*M) or DEX (10 *μ*M) were added 1 h prior to 1 nM CRH treatment. Cells were incubated for another 24 h.

### 2.6. Cell Viability

XTT assays were used to investigate the toxic effects of ADM on HaCaT and HMC-1 cells. After treatment with ADM (1, 10, 30, and 100 *μ*M) for 24 h, 50 *μ*l of XTT solution was added and incubated for 4 h. Absorbances were measured at 450 nm (reference wavelength 650 nm) using a microplate reader (Molecular Devices, CA, USA). Cell viabilities were expressed as percentages of normal controls.

### 2.7. Enzyme-Linked Immunosorbent Assay (ELISA)

Skin and spleen tissues were homogenized with ice-cold tissue extraction reagent (Thermo Fisher Scientific, Vienna, Austria) and centrifuged at 10,000 ×g for 20 min, and supernatants were collected. The levels of IL-4, IL-13, TNF-*α*, IFN-*γ*, and VEGF in skin tissues, levels of TNF-*α*, IFN-*γ*, IL-4, and IL-12 in spleen tissues, and levels of IgE in serum samples were measured using commercial ELISA kits. The levels of TARC/CCL17, MDC/CCL22, IL-6, and ICAM-1 in HaCaT cell culture media and VEGF in HMC-1 cell culture media were determined using commercial kits. Absorbances were measured at 450–550 nm using an automated microplate reader (Molecular Devices, Sunnyvale, CA, USA).

### 2.8. Western Blot Analysis

Skin tissues were homogenized with an ice-cold tissue extraction reagent, centrifuged at 10,000 ×g for 20 min, and supernatants were collected. On the other hand, HaCaT cells were washed with ice-cold 1X PBS, lysed with RIPA lysis buffer containing protease and phosphatase inhibitors (Atto, Tokyo, Japan), and lysates were centrifuged at 8000 ×g for 15 min, and supernatants were collected. Amounts of proteins in samples were determined using Bradford protein assay reagent (BioRad, CA, USA). Proteins (25–50 *µ*g) were separated by 10–12% SDS-PAGE electrophoresis and transferred to polyvinylidene difluoride membranes (Merck Millipore, Carrigtwohill, Ireland), which were blocked with 5% skim milk in 1X PBS for 2 h at room temperature and incubated with the primary antibodies followed by secondary antibody horseradish peroxidase-conjugated anti-IgG. Proteins were detected by enhanced chemiluminescence (BioRad, CA, USA), and band intensities were quantified using GelPro V3.1 software (Media Cybernetics, MD, USA).

### 2.9. Statistical Analysis

The analysis was conducted using the Student's *t* test for unpaired experiments. Results are presented as the mean ± standard deviation (SD) of at least three independent experiments, and statistical significance was accepted for *P* values < 0.05.

## 3. Results

### 3.1. Effects of ADM on AD-Like Symptoms in DNCB-Induced ICR Mice

**Figure 2:**
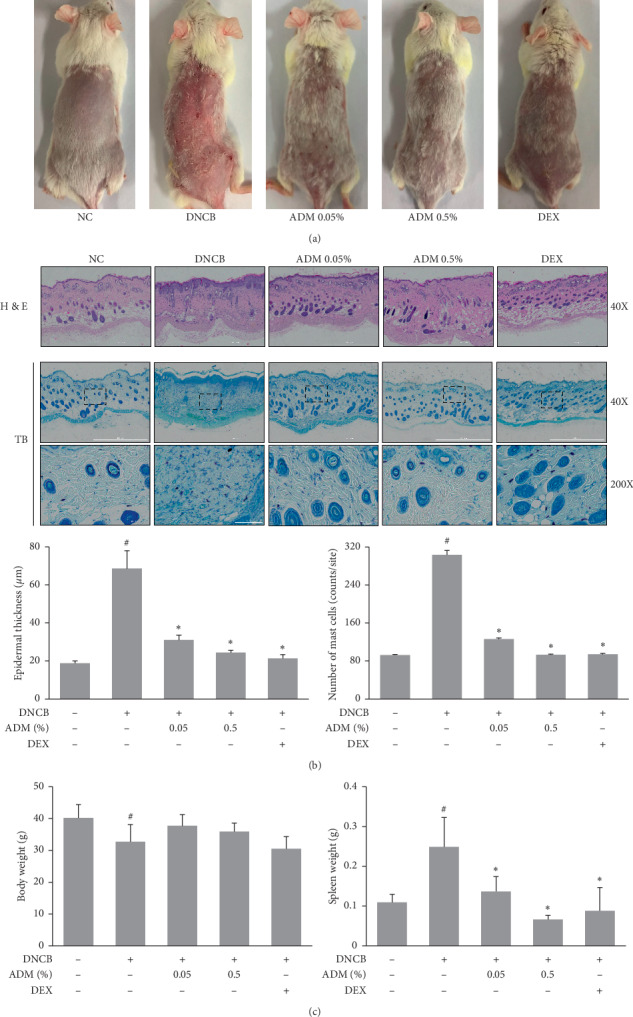
Effects of ADM on AD-like symptoms in DNCB-induced ICR mice. (a) Representative clinical features. (b) Histological analyses: hematoxylin and eosin (H&E) or toluidine blue (TB) staining. (c) Body and spleen weights were measured. Results are presented as mean ± SD (*n* = 5 per experiment). ^#^*P* < 0.05 vs. the NC group; ^*∗*^*P* < 0.05 vs. the DNCB group.

To investigate the effects of ADM on DNCB-induced AD-like symptoms, we evaluated macroscopic signs and morphological changes in mice treated with or without ADM. The DNCB group showed increases in clinical skin symptoms (erythema and dryness) as compared with the NC group. However, the topical administration of ADM significantly reduced these symptoms (Figure 2(a)). Histological observations confirmed morphological changes, such as hyperkeratosis and immune cell infiltration, in skin lesions (Figure 2(b)). Treatment with ADM significantly decreased the epidermal thicknesses and mast cell infiltration into lesioned skin as compared with the DNCB group. We measured body and spleen weights to evaluate general health and immune statuses. DNCB treatment led to a significant reduction in body weights, but ADM treatment moderately increased body weights as compared with DNCB-treated mice. In addition, treatment with ADM also significantly reduced DNCB-induced increases in spleen weights (Figure 2(c)).

### 3.2. Effects of ADM on Skin/Spleen Cytokines and Serum IgE Levels in DNCB-Induced ICR Mice

**Figure 3:**
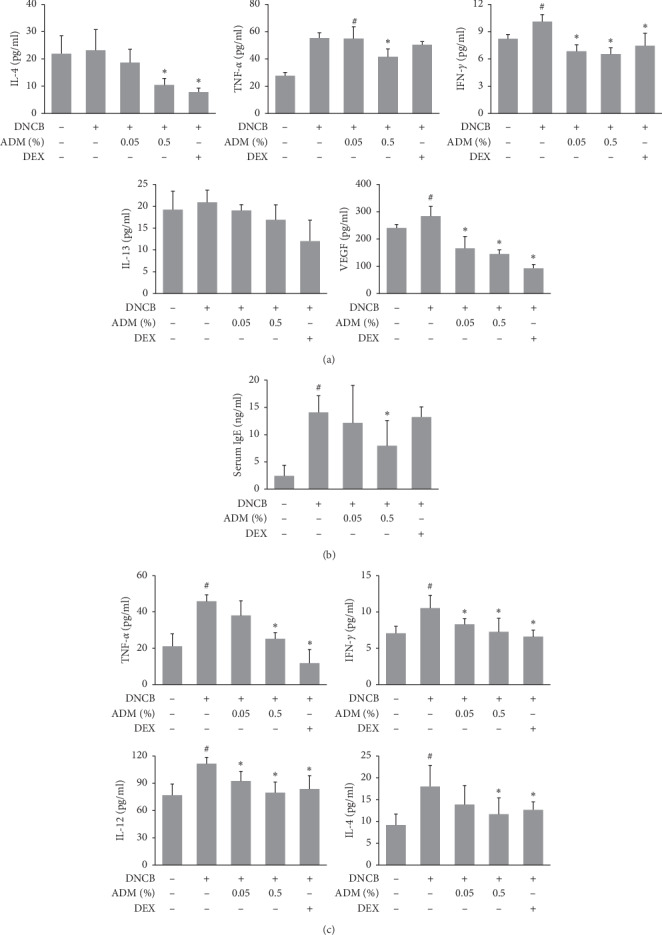
Effects of ADM on skin cytokines and serum IgE levels in DNCB-induced ICR mice. (a) Levels of IL-4, IL-13, TNF-*α*, IFN-*γ*, and VEGF in skin were determined using commercial ELISA kits. (b) Serum IgE levels were determined by ELISA. (c) Levels of TNF-*α*, IFN-*γ*, IL-4, and IL-12 in spleen were determined using commercial ELISA kits. Results are presented as mean ± SD (*n* = 5 per experiment). ^#^*P* < 0.05 vs. the NC group; ^*∗*^*P* < 0.05 vs. the DNCB group.

To assess the effects of ADM on skin inflammation, levels of cytokines in skin lesions were assessed by ELISA. The levels of TNF-*α*, IFN-*γ*, and VEGF were significantly increased in DNCB-treated mice (Figure 3(a)), but treatment with ADM significantly reduced these increases. DNCB did not significantly alter the expressions of IL-4, but ADM treatment significantly decreased levels of IL-4 in skin lesions. Serum levels of IgE (a typical biomarker of allergic diseases, including AD) were also measured. DNCB treatment increased serum IgE levels, but this increase was also reduced by ADM (Figure 3(b)). Moreover, in spleen tissues, the levels of Th1/Th2-related cytokines, including TNF-*α*, IFN-*γ*, IL-12, and IL-4, were upregulated significantly in the DNCB group, which were reduced by the ADM treatment (Figure 3(c)).

### 3.3. Effects of ADM on HPA Axis Hormones in DNCB-Induced ICR Mice

**Figure 4:**
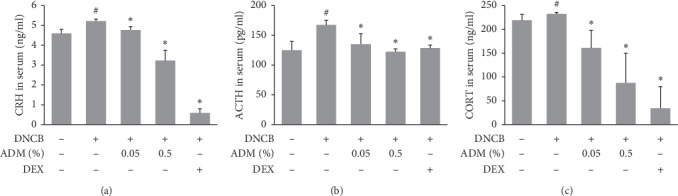
Effects of ADM on HPA axis hormones in DNCB-induced ICR mice. Serum was isolated from each group of mice after sacrifice, and serum levels of CRH, ACTH, and CORT were measured using commercial ELISA kits. Results are presented as means ± SDs (*n* = 5 per experiment). ^#^*P* < 0.05 vs. the NC group; ^*∗*^*P* < 0.05 vs. the DNCB group.

Previous studies have shown that DNCB can activate the HPA axis and induce psychological stress in mouse models [16]. In this study, we evaluated the effects of ADM on CRH, ACTH, and CORT protein levels in DNCB-induced ICR mice.  Figure 4 shows that DNCB treatment significantly increased the serum levels of CRH, ACTH, and CORT, but subsequently, the ADM treatment significantly inhibited these increases in HPA axis hormones.

### 3.4. Effects of ADM on Neuropathological Changes of Hippocampal CA1 Neurons

**Figure 5:**
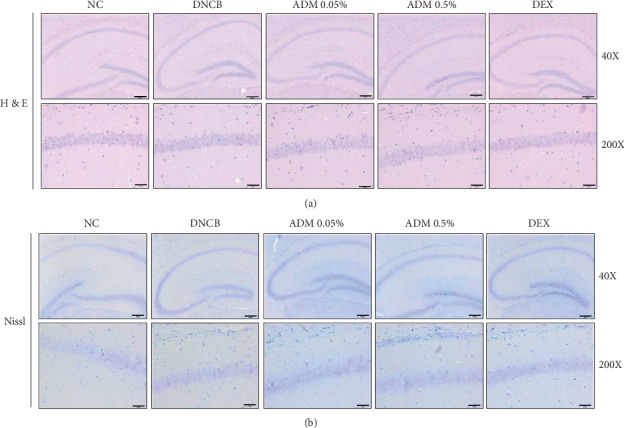
Effects of ADM on neuropathological changes of hippocampal CA1 neurons in DNCB-induced ICR mice. Histological analyses: hematoxylin and eosin (H&E) or Nissl staining.

To evaluate the neuroprotective effect of ADM on DNCB-induced ICR mice, we investigated the morphological changes of neuronal cells in the hippocampal CA1 region. In the NC group, neuronal cells were intact, were well-arranged morphologically, and had abundant cytoplasm and clear nuclei (Figure 5). DNCB treatment did not induce morphological changes of shrunken cytoplasm and nucleic degeneration in neuronal cells. ADM 0.05% and 0.5% group also showed no damage to the hippocampus CA1 region (Figure 5).

### 3.5. Effects of ADM on Proinflammatory Factor Production in TI-Stimulated HaCaT Cells

**Figure 6:**
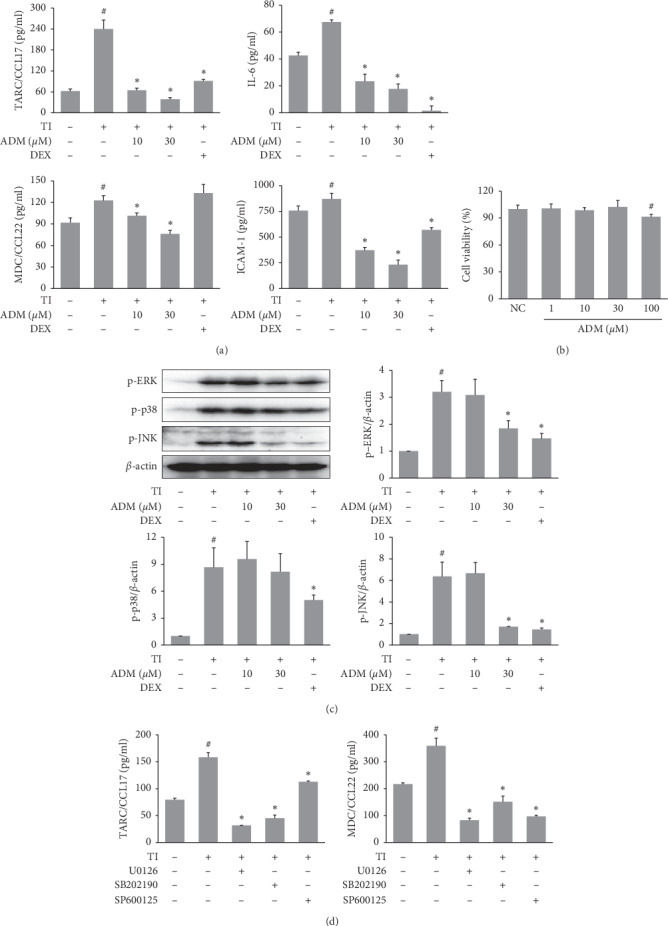
Effects of ADM on proinflammatory factor production and MAPK activation in TI-stimulated HaCaT cells. HaCaT cells were pretreated with ADM (10, 30 *μ*M) for 1 h and then stimulated with TI (TNF-*α* plus IFN-*γ*, 10 ng/ml each) for 24 h (a) TARC/CCL17, MDC/CCL22, IL-6, and ICAM-1 levels in culture supernatants were determined using commercial ELISA kits. (b) Effects of ADM on cell viability were assessed using an XTT assay. HaCaT cells were treated with ADM for 24 h at the indicated concentrations. Effects of ADM MAPK activation in TI-stimulated HaCaT cells HaCaT cells were pretreated with ADM (10, 30 *μ*M) for 1 h and then stimulated with TI for 30 min. (c) Protein expressions of p-ERK, p-p38, and p-JNK were assessed by western blot, and band intensities were normalized versus *β*-actin. (d) HaCaT cells were pretreated with U0126, SB202190, and SP600125 (10 *μ*M) for 1 h and then stimulated with TI for 24 h The TARC/CCL17 and MDC/CCL22 levels in the culture supernatants were determined using commercial ELISA kits. Results are presented as mean ± SD (*n* = 3 per experiment). ^#^*P* < 0.05 vs. normal controls; ^*∗*^*P* < 0.05 vs. TI-treated cells.

Since ADM was found to ameliorate AD-like symptoms in our DNCB-induced mouse model, we investigated the mechanism involved. Epidermal keratinocytes might produce proinflammatory mediators, which are involved in AD-associated skin inflammation. Therefore, we investigated the effects of ADM on the TI-induced expressions of inflammatory factors in HaCaT cells. TI stimulation significantly induced the protein expressions of TARC/CCL17, MDC/CCL22, IL-6, and ICAM-1, and these inductions were significantly inhibited by ADM pretreatment without any cytotoxic effects (Figures 6(a) and 6(b)).

### 3.6. Effects of ADM on MAPK Activation in TI-Stimulated HaCaT Cells

We also examined the effects of ADM on MAPK signaling pathways in TI-stimulated HaCaT cells. TI was found to significantly induce the phosphorylation of ERK, JNK, and p38 MAPK (Figure 6(c)), and pretreatment with ADM significantly reduced then TI-induced phosphorylation. The inhibitors, U0126, SB202190, and SP600125, which are inhibitors of ERK, p38, and JNK, respectively, were used to confirm the role of the MAPK signaling pathways in TI-induced inflammation. As shown in Figure 6(d), treatment with these inhibitors reduced the production of TARC/CCL17 and MDC/CCL22 significantly in TI-stimulated HaCaT cells.

### 3.7. Effects of ADM on Proinflammatory Cytokine Production in CRH/SP-Stimulated HMC-1 Cells

**Figure 7:**
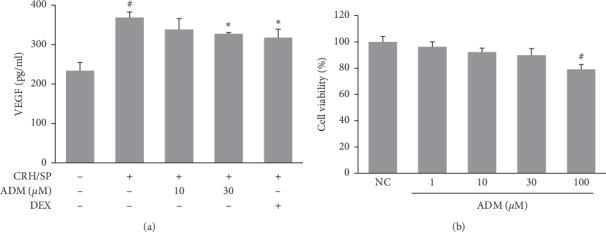
Effects of ADM on proinflammatory factor expression in CRH/SP-stimulated HMC-1 cells. HMC-1 cells were incubated with 10 *μ*M SP for 48 h. Then, ADM (10, 30 *μ*M) or DEX (10 *μ*M) were added 1 h prior to 1 nM CRH treatment. Cells were incubated for another 24 h (a) VEGF level in culture supernatants was determined using commercial ELISA kits. (b) Effects of ADM on cell viability were assessed using an XTT assay. HMC-1 cells were treated with ADM for 24 h at the indicated concentrations. Results are presented as mean ± SD (*n* = 3 per experiment).^#^*P* < 0.05 vs normal controls; ^*∗*^*P* < 0.05 vs. CRH/SP-treated cells.

We used CRH/SP-stimulated HMC-1 cells as an in vitro model of AD-associated stress to examine the effects of ADM. Stimulation with CRH/SP significantly increased the secretion of VEGF by HMC-1 cells, but pretreatment with ADM treatment significantly inhibited this increase without affecting the cell viability (Figure 7).

## 4. Discussion

Topical corticosteroid application is the most common and effective approach for treating AD [12]. However, although corticosteroids have potent anti-inflammatory properties, they serious side effects, which include skin atrophy, skin barrier dysfunction, and immunosuppression [17], and these create an urgent need for new drugs with fewer side effects. Arctigenin is the principal compound in Forsythiae Fructus and has been reported to suppress B-cell significantly and T-cell-mediated allergic inflammation and proinflammatory cytokine production in vivo and in vitro [18]. Hederagenin is a triterpene from Lonicerae Flos that has been demonstrated to inhibit inflammatory cytokine production in lipopolysaccharide-stimulated RAW 264.7 cells and decrease skin thickening, inflammatory cell infiltration, and mast cell degranulation in a carrageenan-induced mouse model of edema [19]. Baicalein is a well-known flavonoid from Scutellariae Radix that has been demonstrated to significantly improve AD-symptoms by inhibiting proinflammatory cytokine and mast cell infiltration into skin lesions in NC/Nga mice [20]. In this study, we demonstrated that a natural compound mixture (ADM), containing arctigenin, hederagenin, and baicalein, possessing antiatopic activities in both in vivo and in vitro models.

The topical application of DNCB to ICR mice was shown to induce AD-like skin symptoms, such as erythema, pruritus, and dryness [21]. Our results showed that these symptoms were significantly reduced by ADM treatment. In addition, the topical application of ADM also alleviated DNCB-induced epidermal thickening and mast cell infiltration into dorsal lesions. Interestingly, DNCB application also induced enlarged spleen in ICR mice. ADM treatment remarkably reduced this effect of DNCB without significant alteration in body weight in comparison with the normal control group. The spleen is one of the most important immune organs, and spleen enlargement is usually associated with infection and inflammatory diseases [22]. In the present study, DNCB induced AD-like symptoms and increased spleen weights; in contrast, ADM treatment suppressed these phenomena.

We also examined the effects of ADM on inflammatory cytokine production and IgE secretion in the DNCB-induced mice model. Previous studies have demonstrated that an elevated serum IgE level is a hallmark of AD [23]. When IgE binds to its receptors on mast cells, it prompts them to release proinflammatory mediators [24]. We found that ADM significantly reduced serum IgE in our mouse model. Interestingly, previous studies have reported that the whole extracts of Lonicerae Flos or Scutellariae Radix did not influence the serum IgE in murine models of AD [12, 15] but that a mixture of their main compounds (hederagenin and baicalein) significantly inhibited serum IgE increases in DNCB-induced mice. This is possible that the ADM contained sufficient amounts of active compounds to exhibit an inhibitory effect on the IgE level. AD is associated with increased IgE levels as well as the upregulation of Th1 and Th2 cytokines in skin lesions [25]. The results from a clinical trial which revealed increased Th1 and Th2 responses to the allergen in AD children [26]. DNCB, a well-known skin sensitizer, can induce Th1 responses, but circulating DNCB-specific antibodies are generated, and a Th2 response can also be induced [27]. The Th2 cytokines, IL-4, and IL-13 play important roles in increasing the IgE level and recruiting mast cells. The Th1 cytokines, IFN-*γ*, exacerbate inflammation in AD-like skin lesions by activating macrophages [28]. TNF-*α* binding to its receptors on immune cells and keratinocytes prolong the inflammation in AD, and VEGF enhances vascular permeability and induces vascular endothelial cell proliferation, which is associated with inflammatory processes in AD lesional skin [11]. In the present study, ADM (0.5%) inhibited IL-4, IFN-*γ*, TNF-*α*, and VEGF production in skin lesions. The inhibitory effects of ADM on the Th1 and Th2 immune responses and the proinflammatory cytokines in skin lesions may be associated with a reduced epidermal thickness and mast cell infiltration.

The effects of ADM on proinflammatory mediator secretion in HaCaT cells (a human keratinocyte cell-line) were also investigated. Epidermal keratinocytes play an important role in the pathogenesis of AD by secreting a variety of chemokines and cytokines. Several studies have reported that TARC/CCL17 and MDC/CCL22 are highly expressed chemokines in keratinocytes in the presence of AD [4]. These chemokines are required for the recruitment of Th2 cells to inflammatory skin lesions, and Th2 cell infiltration increases the levels of proinflammatory factors and maintains a chronic inflammatory status in lesioned skin. Keratinocytes from AD patients have also been shown to contain high levels of various cytokines and adhesion molecules, such as IL-6 and ICAM-1 [29, 30]. In the present study, we examined the effects of ADM on protein expression of these inflammatory factors in HaCaT cells. ADM treatment suppressed TNF-*α*/IFN-*γ*- (TI-) induced TARC, MDC, IL-6, and ICAM-1 expression in HaCaT keratinocytes, which suggests ADM might exert a therapeutic effect by downregulating levels of AD-related factors in keratinocytes. MAPK signaling pathways are activated in response to TI stimulation, leading to the secretions of proinflammatory factors by HaCaT cells. Treatment with p38, ERK, or JNK inhibitor significantly reduced the mRNA and protein expressions of TARC and MDC in TI-stimulated HaCaT cells [31], whereas, in the present study, TI stimulation activated the phosphorylation of p38, ERK, and JNK in HaCaT cells. However, pretreatment with ADM suppressed the phosphorylation of ERK and JNK significantly, suggesting that the inhibitory effects of ADM on TI-induced proinflammatory mediator upregulation might be mediated by blocking the ERK and JNK MAPK signaling pathways in HaCaT cells.

Recent studies have indicated a bidirectional relationship exists between AD and psychological stress and that stress can exacerbate skin symptoms in AD patients, which in turn might trigger psychological stress leading to various mental disorders, including anxiety, depression, and attention-deficit hyperactivity disorder (ADHD) [7, 32]. A study on NC/Nga mice demonstrated that DNCB application not only induced AD-like symptoms but also dysregulated the dopamine and noradrenaline levels (ADHD phenomena) and HPA axis activation [16]. Similarly, we observed HPA axis hyperactivation in DNCB-induced ICR mice. In contrast, treatment with ADM significantly reduced DNCB-induced upregulation of HPA axis hormones, including CRH, ACTH, and CORT. The proinflammatory cytokines have been shown to stimulate the HPA axis activity [33]. Hyperactivation of the HPA axis helps upregulate Th2 cytokines, such as IL-4 and IL-5, as well as Th1 cytokines, such as IFN-*γ* [34]. When ADM (0.5%) was applied directly to the skin, it inhibited the production of Th1 and Th2 cytokines and inhibited the activation of the HPA axis. These results are consistent with a previous report showing that melatonin has anti-inflammation effects by regulating the HPA axis via the skin [16]. On the other hand, more research will be needed to analyze how the ADM influences the relationship between the HPA axis and the Th1/Th2 responses. Further study will be needed to determine if the topical application of ADM regulates the HPA axis directly or indirectly.

The hippocampus is an important region for the stress response and is vulnerable to corticosteroid-induced neuronal damage [35]. A previous study reported that hippocampal inflammation was induced in a DNCB-induced AD-like mouse model [36]. High concentrations of CORT can cause hippocampal neuronal damage, including morphological changes, decreased neurogenesis, and apoptosis [9]. Skin damage caused by UV irradiation increased the levels of CORT in the serum and subsequently decreased hippocampal neurogenesis and caused depression-like behavior [37]. In our study, DNCB treatment increased the level of serum CORT in ICR mice; however, we did not observe neuronal cell defects or apoptosis in the hippocampus. These contrary results may have been due to the short duration of the DNCB treatment or varied responses among different mouse strains.

The HPA axis is responsible for modulating the inflammatory responses, among which CRH plays an important role in AD. CRH has proinflammatory effects through mast cells. Mast cells express the CRH receptors, and CRH enhances the production of VEGF. Therefore, AD can be triggered or exacerbated [38, 39]. In addition to CRH, ACTH and CORT are involved in the homeostatic skin functions. ACTH is involved in melanin production, and CORT exerts negative effects on the skin barrier function and the stratum corneum integrity [34, 40]. In this study, the topical application of ADM not only inhibited mast cell infiltration and VEGF production in skin lesions but also reduced the serum CRH level in a dose-dependent manner. Therefore, to determine if ADM inhibits CRH-induced VEGF production in mast cells, an in vitro assay was also performed using HMC-1 cells. Human mast cells (HMC-1 cells) stimulated with CRH and SP were used to produce an in vitro model of AD-associated stress. Mast cells play a critical role in allergic diseases, including AD, and are involved in the elicitation of immune response by secreting a variety of inflammatory cytokines, proteases, and vasoactive molecules [38]. The neuropeptide SP has been reported to activate mast cells to secrete histamine and proinflammatory mediators by binding to neurokinin-1 receptors on cutaneous mast cells [41]. Interestingly, the expression of SP is also increased in response to stress [42]. Previous studies have indicated CRH or SP can induce mast cell activation, but that in combination, CRH and SP enhance inflammatory responses in mast cells [10, 43]. We observed that HMC-1 cells stimulated with SP and CRH showed increased levels of VEGF and that this increase was significantly suppressed by ADM pretreatment. VEGF induced the vascular permeability and had an angiogenic effect. Moreover, its serum levels were correlated with the severity of AD [11, 44–46]. These results suggest that ADM might be a potential candidate for the treatment of stress-exacerbated AD.

## 5. Conclusion

The topical application of ADM alleviates AD-like symptoms and HPA axis activation in DNCB-induced ICR mice. Pretreatment with ADM inhibited the TNF-*α*/IFN-*γ* induced inflammatory factor production by inhibiting ERK and JNK MAPK signaling pathways in HaCaT keratinocytes and inhibited inflammatory cytokine secretion by CRH/SP-stimulated HMC-1 cells. Our results suggest that ADM is a potential candidate for the treatment of AD.

## Data Availability

All data used to support this study are included within the article.

## Abbreviations

DNCB: 2,4-Dinitrochlorobenzene
CRH: Corticotropin-releasing hormone
TARC: Thymus and activation-regulated chemokine
MDC: Macrophage-derived chemokine.
